# Reliability of pelvic floor muscle surface electromyography (sEMG) recordings during synchronous whole body vibration

**DOI:** 10.1371/journal.pone.0251265

**Published:** 2021-05-18

**Authors:** Daria Chmielewska, Grzegorz Sobota, Paweł Dolibog, Patrycja Dolibog, Agnieszka Opala-Berdzik

**Affiliations:** 1 Electromyography and Pelvic Floor Muscles Laboratory, Department of Physical Medicine, Institute of Physiotherapy and Health Sciences, The Jerzy Kukuczka Academy of Physical Education, Katowice, Poland; 2 Department of Human Motor Behavior, Institute of Sport Science, The Jerzy Kukuczka Academy of Physical Education, Katowice, Poland; 3 Department and Faculty of Medical Biophysics, Medical University of Silesia, Katowice, Poland; 4 Department of Physiotherapy in Internal Diseases, Institute of Physiotherapy and Health Sciences, The Jerzy Kukuczka Academy of Physical Education, Katowice, Poland; Universita degli Studi di Milano, ITALY

## Abstract

The primary aim of the study was to assess intraday and interday reliability of surface electromyography (sEMG) reflex activity of the pelvic floor muscles during synchronous whole-body vibration (S-WBV) of two intensities (30Hz/2mm; 40Hz/4mm) using band-stop filter and high-pass filter signal processing. The secondary aim of the study was to assess intraday and interday (test-retest) reliability of sEMG obtained from maximal voluntary contraction (MVC) test. We evaluated the intraday reliability of sEMG recordings obtained during sessions 1 and 2 performed on the same day. The sessions consisting of maximal voluntary pelvic floor muscle contraction and synchronous vibration sets with 1-hour rest in-between sessions 1 and 2 in healthy nulliparous women. The next intraday reliability was evaluated between the results of sessions 3 and 4 performed on the same day but followed at an interval of 4 weeks. to include the entire menstrual cycle. The interday reliability was determined based on the results of sessions 1 and 3 using the intraclass correlation coefficient (ICC 3,3). The intraday ICCs for band-stop filtered mean and median sEMG frequency and mean normalized sEMG_RMS_ amplitude of the 30Hz/2mm (ICC = 0.89–0.99) and 40Hz/4mm vibration (ICC = 0.95–0.99) indicated substantial reproducibility. The intraday reliability of high-pass filter at 100-450Hz for these parameters was also substantial (30Hz/2mm ICC of 0.92 to 0.98; 40Hz/4mm ICC of 0.88 to 0.98). The interday reliability (session 1 vs. session 3) of the mean normalized sEMG_RMS_ amplitude for band-stop filtered means of 40 Hz/4mm and 30Hz/2mm vibration recordings was substantial (ICC = 0.82 and 0.93). However, ICCs of the mean and median frequency were indicative of fair reliability (ICC of 0.43 to 0.59). The interday reliability of mean normalized sEMG_RMS_ amplitude for high-pass filter at 100-450Hz was substantial (30Hz/2mm ICC of 0.90; 40Hz/4mm ICC of 0.73) for the 30Hz/2mm S-WBV and moderate (ICC = 0.73) for the 40/4mm S-WBV. The ICCs for mean and median sEMG frequency ICCs indicated slight to fair reproducibility (ICC of 0.16 to 0.56). The intraday reliability of the strongest MVC contraction and average MVC turned out substantial (ICC = 0.91–0.98). The interday reliability coefficients of the strongest MVC contraction and average MVCs were 0.91 and 0.82, respectively. Concluded, the intraday reliability proved satisfactory for all variables; however, the interday comparison showed sufficient ICC levels only for the mean amplitude. We therefore recommend this parameter should be used when analyzing PFM sEMG recorded during vibration. ICCs of the mean and median frequency for both signal processing methods were indicative of insufficient reliability and did not reach the threshold for usefulness. Our study showed similar reliability of PFM sEMG during S-WBV in case of the two filtering methods used.

## Introduction

There is a growing interest of researchers and practitioners in the effect of whole body vibration (WBV) exercise in training [1]. WBV has beneficial effects on neuromuscular performance as it improves the strength and power of muscles [2]. WBV has been used in clinical rehabilitation [3, 4] and preventive medicine. The most popular is sinusoidal vibration, which is applied through a vibrating surface. Some models apply the vibration in a side-alternating way (sequentially to the right and left foot), others transmit vibration via a plate that causes synchronic up and down movement of the right and the left foot–synchronous whole body vibration (S-WBV) [5]. Mechanical vibration of a human skeletal muscle induces a tonic vibration reflex [6] via activation of a polysynaptic pathway terminating on tonic alpha motor neurons [7] with the involvement of neural mechanisms associated with spinal reflexes, muscle tuning and central motor command [8].

Pelvic floor muscles (PFM) are striated muscles that include muscle spindles [9] therefore being capable of responding to mechanical vibration. Lauper et al. [10] were among the first to mention an increase in sEMG activity of the PFM over baseline activity in standing in postpartum and healthy control women—depending on vibration intensity. These beneficial effects were demonstrated in response to sinusoidal (side-alternating vibration) and stochastic whole-body vibration, with the superiority of stochastic resonance vibration (6–12 Hz); the effects were more pronounced in the postpartum group with weakened PFM contractions [10]. Other authors found that PFM sEMG activity during WBV was maximized in healthy individuals [11, 12]. Literature also provides data to support satisfactory intra-session retest reliability of PFM sEMG parameters during rest and MVC sEMG analysis in healthy and women with PFM dysfunction [13, 14]. However, no analyses have been carried out of PFM sEMG reliability that would incorporate the risk of vibration-induced motion artifacts.

Considering the risk of motion artifact and reflex activity contributions to EMG amplitude, surface EMG activity recording during vibration exercise is difficult [15, 16]. Sebik et al. [17] observed motion artifact-contaminated sEMG signals and, additionally, motor unit synchronization at the vibration frequency of WBV platform. Muscle activity measured during WBV can be overestimated when the spikes are not deleted [16]. Filtering, on the other hand, may limit the identification of synchronous motor unit activity phase-locked to the vibration frequency of the WBV platform [17]. Motion artifacts can be eliminated using a high-pass or band-stop filter. Sebik et al. [17] developed a method in which sEMG signals were filtered at 80 Hz high-pass level and then full-wave rectified. Hazell et al. [18] only retained the high frequency signals (100–450 Hz); thus, all noise caused by the frequency of the vibration platform was removed. Digital band-stop filtering is based on the elimination of large, vibration-induced motion artifacts and multiple harmonics from raw EMG data [19]. Lienhard et al. [20] confirmed that sEMG processing methods, e.g., spectral linear interpolation or band-stop filter centered at the vibration frequency, should be applied during whole-body vibration to delete the artifacts in the sEMG signals of the vastus lateralis and soleus muscles. On the other hand, Ritzmann et al. [21] did not find any significant amount of motion artifacts during sinusoidal WBV. The authors hypothesized that periodic spikes in EMG recordings during whole body vibration were stretch reflexes induced in leg. They suggested it was possible to use EMG data recorded during WBV without applying additional filters because the contribution of motion artifacts seemed to be insignificant.

The present study focuses on testing the intraday and interday reliability of PFM sEMG during synchronous whole body vibration (S-WBV) of two intensities using two signal processing methods described in literature. A wider insight into PFM sEMG response to vibration gained from measurement reliability assessment may help interpret the effects of vibration on voluntary and reflexive PFM activity in healthy women and women with pelvic floor dysfunction.

The primary aim of the study was to assess intraday (between two sessions within the same day) and interday (test-retest) reliability of reflex sEMG activity of the pelvic floor muscles during 60 seconds of synchronous whole-body vibration (S-WBV) of two intensities (30Hz/2mm; 40Hz/4mm). The secondary aim of the study was to assess intraday and interday (test-retest) reliability of sEMG obtained from MVC test. In our experiment, sEMG activity was processed using two filtering methods (band-stop filter, high-pass at 100-450Hz filter). We hypothesized that the reliability of sEMG signals recorded during vibration might differ depending on the signal filtering method.

## Materials and methods

The study protocol was approved by the Bioethics Committee at the Academy of Physical Education in Katowice, Poland (1/2017). The recruited women received comprehensive information on the study aim and methods, and gave their informed written consent to participate as required by the Declaration of Helsinki". The present study constitutes a phase of a project designed to determine the effect of whole body vibration on reflexive PFM activity and voluntary contraction (ACTRN12618000531213).

### Participants and setting

The participants were recruited out of students of our Academy by the flyers which contained information about the study aim and procedure. The flyers were posted inside the Academy building. The recruitment process lasted from February 2019 to June 2019. The recruitment was carried out by a person not involved in the investigations. The participants were included based on the following inclusion criteria: nulliparous women without PF dysfunction, regular menstruation, good general health and no history of previous vibration platform exercises. Exclusion criteria included history of (or current) stress urinary incontinence, pregnancy, childbirth(s), pelvic surgery, diabetes, hypertension, neurological abnormalities, urinary tract infection, elevated temperature, practicing professional sport, spinal pain, pelvic organ prolapse, unhealed fracture, nephrolithiasis. Ten healthy adult women were invited to participate. One woman was excluded as she did not meet the inclusion criteria; another one could not participate in the second measurement session due to urinary tract infection. Ultimately, 8 healthy nulliparas entered the study (Table 1). During an introductory session, all recruited subjects were instructed on the correct maximal voluntary contraction of the PFMs while observing sEMG signals on the computer monitor.

**Table 1:** Characteristics of study participants.

Characteristics	group n = 8		
	mean ± SD or n (%)	min.	max
Age [years]	24.2 ± 2.9	20	30
Body weight [kg]	64.1 ± 10.2	45	75
Height [cm]	167 ± 4.6	157.5	176
BMI [kg/m^2^]	22.8 ± 2.8	18.4	26.6
Current oral contraception users			
No	5 (62.5%)		
Yes	3 (37.5%)		

To consider the study sample representative of a larger population, the sample size was determined based on Walter et al. recommendations [22]. We assumed that sample size calculation included the minimal acceptable reliability coefficient (ICC = 0.6), expected reliability (ICC = 0.9), three repetitions of sEMG recordings during vibration of 30Hz/2mm and 40Hz./4mm, separately, the probability of a type I error alfa rate of 0.05, maximum acceptable value for a type II error beta rate of 0.20. It was calculated that the required minimum sample size was 8 participants.

The women enrolled in the study reported for testing to the Electromyography and Pelvic Floor Muscles Laboratory at the Institute of Physiotherapy and Health Sciences, at the Jerzy Kukuczka Academy of Physical Education in Katowice.

### Intervention

#### Study procedure

**Fig 1:**
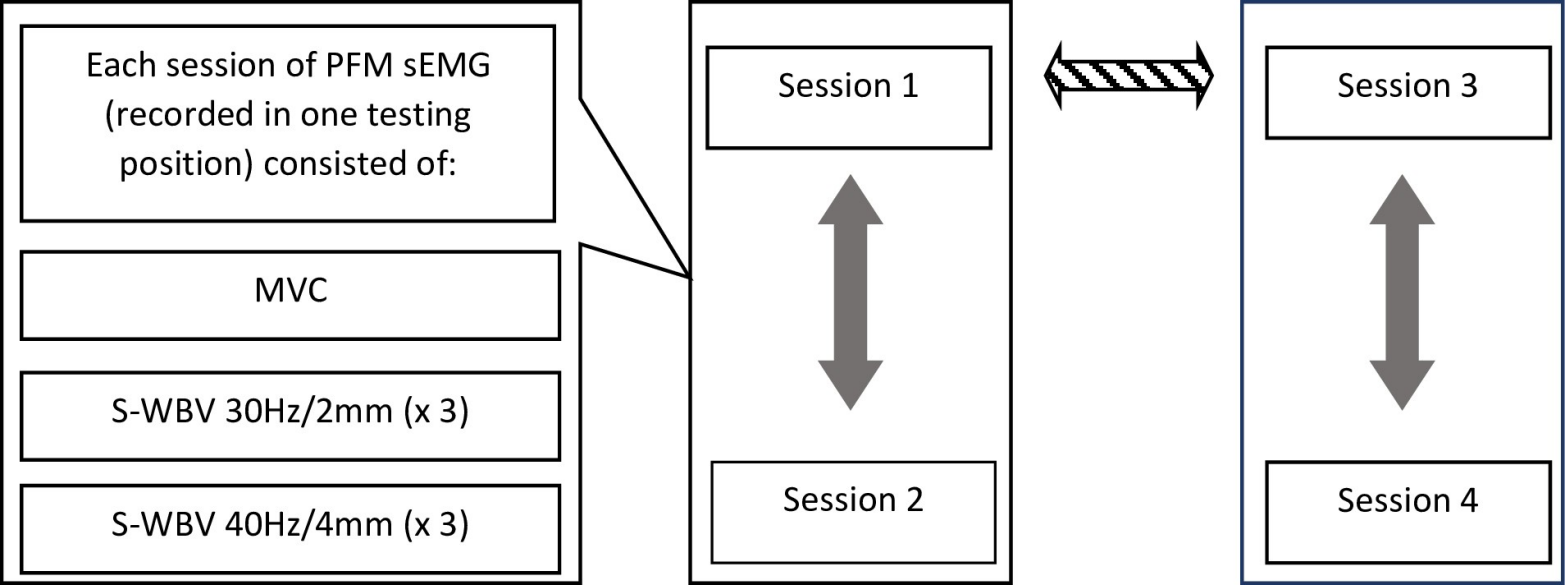
Study procedure of surface electromyography (sEMG) of pelvic floor muscles (PFM) which was performed to analyze intraclass correlation coefficients. Filled arrows indicate intraday reliability (session 1 vs. session 2; session 3 vs. session 4), striped arrow indicates interday reliability (session 1 vs. session 3), MVC–maximal voluntary contraction; S-WBV–synchronous whole body vibration.

The study procedure consisted of four sessions of sEMG recording (Fig 1). Two sessions (session 1 and session 2) were performed on the same day and were separated with one-hour rest to assess intraday reliability. The next two sessions (sessions 3 and 4) were repeated at an interval of 4 weeks to include the entire menstrual cycle. The sEMG recording sessions 3 and 4 were also separated with one-hour rest to test intraday reliability (session 3 vs. session 4). Interday reliability was then analyzed based on session 1 and session 3 (Fig 1). Each of four sessions of sEMG included the recording of: 1/ PFM MVC (three trials), and 2/ reflex PFM activity during S-WBV set (three repetitions of 60-second sEMG recordings during S-WBV with 30Hz/2mm intensities and three repetitions of 60-second sEMG recordings during S-WBV with 40Hz/4mm intensity). Prior to ICC analysis, the values of the sEMG parameters from three repetitions were averaged.

Participants were scheduled for the examination sessions just after menstruation but no later than two days after the end of their menstrual bleeding to assure that they all were in the follicular phase of their cycles. The reexamination was performed following one menstrual cycle in the next follicular phase (no later than two days after the end of the next menstruation) which was in about four weeks. Performing both the examination and reexamination in the same phase of the menstrual cycle allowed to eliminate the cycle-related effect of hormonal fluctuation on PFM activity [23].

The participants were asked not to take up intensive physical exercises 24 hours before each session. Prior to PFM sEMG, they were also asked to empty their bladder. The mode of experiment administration was one-to-one and face-to-face. During each sEMG session the subjects were wearing socks. The recording took place while standing on the vibration platform in semi-squat (with feet hip-width apart; hip and knee flexion 35°) testing position [11] without touching the handrail of the platform. The knee angle was controlled with a goniometer. Each session lasted for a maximum of 2.5h.

#### PFM sEMG recording

Bioelectric potentials generated by the PMFs were recorded with pear-shaped and tapered vaginal probes (Life-Care Vaginal Probe PR-02, Everyway Medical Instruments Co.). This design of vaginal electrode might be less prone to recording motion artifacts than cylindrical probes [24]. Each study participant was equipped with a personal probe. The probe was inserted using a small (5ml) amount of antiallergic lubricant with the sensors positioned laterally in the vagina. Sensor location was marked on the outer portion of the electrode, which allowed checking the correct positioning of the device prior to each recording. The reference electrodes were placed over the right anterior superior iliac spine (ASIS). Simultaneously with the recording of PFM bioelectrical potentials, sEMG of the rectus abdominis, internal abdominal oblique/transverse abdominal muscles was performed (in accordance with SENIAM using bipolar self-adhesive silver/silver chloride electrodes) in order to monitor their coactivation with PFM. The vaginal electrode and surface electrodes remained in the same place within the same examination day and were replaced between days. During each session sEMG device’s cables were taped to the skin to minimize mechanical artifacts. All sEMG recordings were performed by the same investigator.

PFM electrical activity was recorded using Myo Trace 400 (Noraxon U.S.A. Inc.) sEMG with a preamplifier (band pass filter 20Hz-500Hz, Common Mode Rejection Ratio of >100dB at 60Hz, input impedance >100 mega-ohms, amplifier gain 500). A 16-bit analog to digital (A/D) converter with a sampling frequency of 1000Hz.

#### sEMG during PFM maximal voluntary contractions

During each session, the sEMG MVC recording comprised of three 5-second attempts at maximum contraction of the PFM in the testing position, with a 60-second rest in between each attempt.

#### sEMG of PFM reflex activity during whole body vibration

To record reflex activity of PFM the sEMG was performed during S-WBV on a vibration platform (Fitvibe 600, Gymna Uniphy N.V.). The vibration set comprised of three sEMG recordings during vibration with frequency of 30Hz and amplitude of 2mm, and three recordings during vibration with frequency of 40Hz and amplitude of 4mm (applied at random order). Each vibration exposure lasted 60 seconds; a 5-minute rest was allowed between the recordings to eliminate potential PFM fatigue. The participants were not informed about vibration intensity.

### sEMG signal processing

#### sEMG of maximal voluntary contractions signal processing

MVC sEMG data were filtered at 20-450Hz, after filtering signal was rectified and the root mean square value was calculated using a 100 ms sliding window. EMG_RMS_ parameter used to measure the amplitude.

#### sEMG signal processing during vibration

We employed two methods of raw sEMG signal processing: band-pass filter by Hazell et al. [18] and band-stop filter by Abercromby et al. [19]. The data were processed with MATLAB software package (R2017B, The Mathworks, Inc., Natick, MA).

#### Band-stop filter sEMG

In order to delete sEMG signal spikes that might be considered vibration artifacts in fundamental frequency and its harmonics, the sEMG signal was filtered using the filtering regimen by Abercromby et al. [19]. The band-stop filter 17^th^-order Chebyshev type II with a band-stop of ±1Hz, transition band of ± 1.5Hz, minimum band-stop attenuation of 100dB and maximum 0.01dB ripple was used. The filter was centered at the frequency of the fundamental vibration frequency (determined by the platform conditions and checked with the Fast Fourier Transform and signal frequency spectrum) and the harmonics of up to 450 Hz. In all sEMG records, the power line frequency of 50Hz and its harmonics 100 Hz were filtered. The data were full-wave rectified and the root mean square (sEMG_RMS_) was calculated within a 100-ms window.

#### Band-pass filter sEMG

As a separate filtering protocol, we used regimen employed by Hazell et al. [18]. The sEMG signal was band-pass filtered (20–450 Hz); using a dual passed sixth-order Butterworth, it was then filtered between 100 and 450 Hz (high-pass filter). In all sEMG records, the harmonics at 100Hz of power line frequency was filtered. The data were full-wave rectified and the root mean square (sEMG_RMS_) was calculated within a 100-ms window.

### sEMG parameters

Based on the processed signal, the following sEMG parameters were calculated from each sEMG recordings, both from time and frequency domain: the mean of root mean square amplitude value next normalized to the maximal voluntary contraction value to represent the reflex PFM activity (EMG_RMS_ %MVC), the root mean square amplitude value of MVC, and the mean (MNF) and median (MDF) frequencies. The analyses of the median frequency (MDF) and mean frequency (MNF) of EMG signals were based on the Total Power Spectrum which had been determined using Fast Fourier Transformations (FFT).

Reliability of sEMG signals concerning reflex PFM activity during vibration was analyzed based on the means calculated from three sEMG_RMS_ recordings for each of the filtering methods. Analyses of reliability of MVC sEMG_RMS_ amplitude were based on the means of three 5 s MVC contractions and the amplitude from the strongest contraction (the greatest value of three MVC trials).

### Statistical methods

The Shapiro–Wilk test was used to check whether the data had normal distribution. The mean and standard deviation values were calculated for all parameters. Data skewness, kurtosis and modality were also checked.

The mean values of MVC and vibration sEMG variables (sEMG_RMS_ amplitude, mean and median sEMG frequency) were compared with repeated measures ANOVA [repeated factor- four levels: four sessions].

Derived from the ANOVA results of the mean square of error and mean square between subjects, the reliability was assessed by the intraclass correlation coefficient (ICC). ICC estimates and their 95% confidence intervals were calculated based on a mean value of three sEMG recordings (*k* = 3), 2-way mixed-effects, consistency, multiple measurements model ICC (3,3) [25]. Three sEMG recordings were taken from each participant by a single rater during each of four sessions and for two vibration intensities. Next, the three values of sEMG_RMS_ amplitude, mean and median frequency were averaged to produce each data point. The averaging of the sEMG values occurred before the entry into the ICC analysis what gave eight pairs of datapoints. The ICC (3,1) was used to analyze the intraday and interday reliability of the greatest MVC value (*k* = 1). The intraday and interday reliability coefficients of the average value from three MVC measurements (*k* = 3) were analyzed by the ICC (3,3). The following ICC intervals were chosen: a) ≤ 0.10, no reproducibility: b) 0.11–0.40, slight reproducibility; c) 0.41–0.60, fair reproducibility, d) 0.61–0.80, moderate reproducibility; e) 0.81–1.0, substantial reproducibility [26]. The correlation coefficient (CC) was also used. Standard error of measurements (SEM) was calculated ($SEM=SD\sqrt{1-ICC}$), where SD was determined as $\sqrt{SStotal/(n-1)}$) [27]; coefficients of variation (CV%) were calculated by dividing the standard deviation (SD) by the mean value of the sample and multiplication by 100 (%), and minimal differences (MD) as $MD=SEMx1.96x\sqrt{2}$.

In all tests, the level of statistical significance was set at p = 0.05. The tool for data analysis was Statistica, version 13.3.

## Results

**Fig 2:**
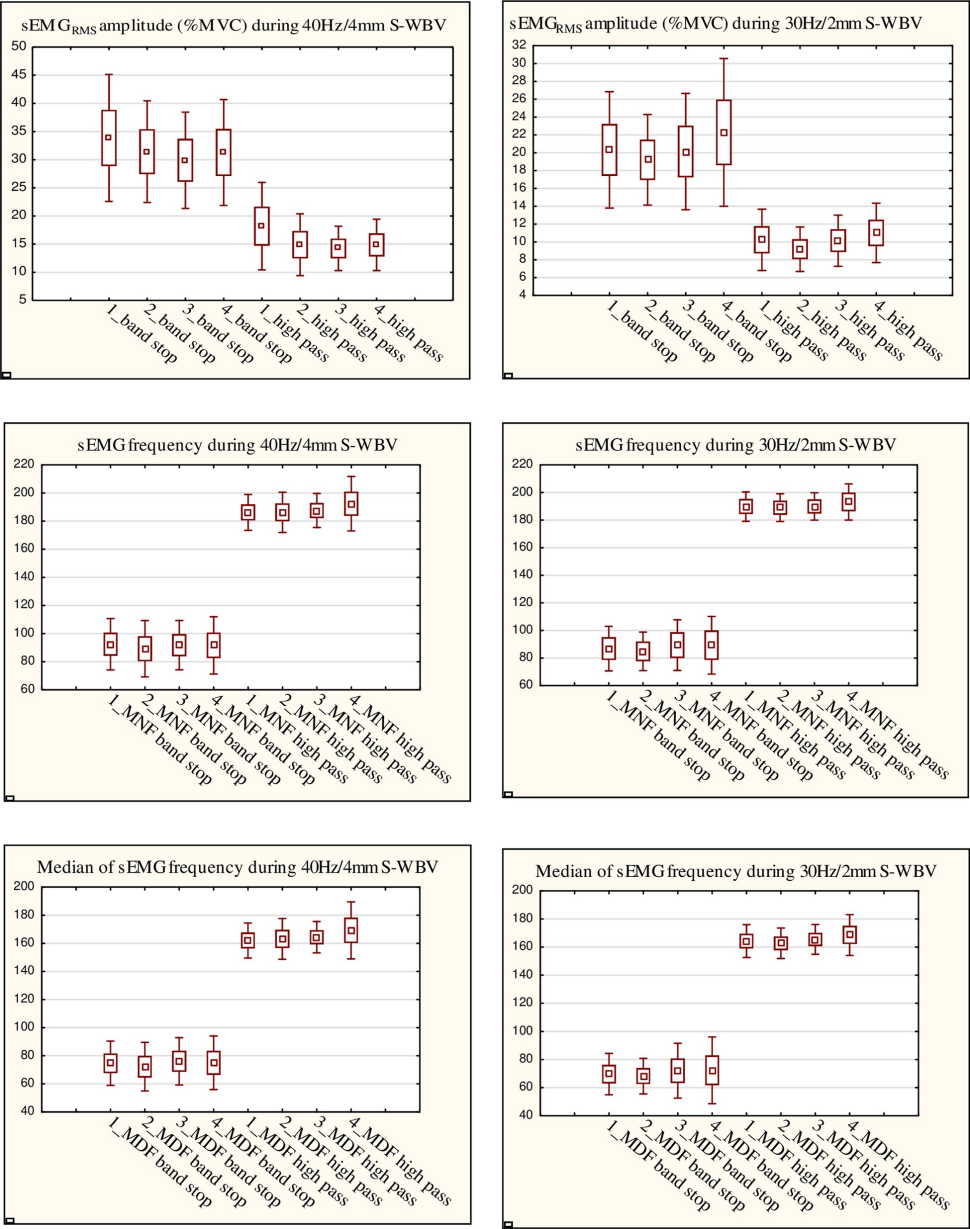
Mean values with standard errors and standard deviations of sEMG parameters acquired from sEMG recordings using vibration of two intensities. 1; 2; 3; 4 -number of session.

The sEMG_RMS_ amplitude, mean and median sEMG frequency (mean ± SD) of the two processing methods during vibration of two intensity are shown in Fig 2.

### Intraday reliability

The ICC for band-stop filtered mean and median frequencies and the mean normalized sEMG_RMS_ amplitude of the 30Hz/2mm vibration indicated substantial reproducibility; the inter-measurement variability was low, i.e., between 3.15% and 15.62%. The intraday reliability of high-pass filter at 100-450Hz was substantial with inter-measurement variability of 2.12% and 15.93% (Table 2).

**Table 2:** Band-stop filtered and high-pass filtered intraday reliability of 30Hz/2mm—whole body vibration sEMG signals from the pelvic floor muscle.

Parameter (unit)	session	mean (±SD)	ICC	95% CI	CV (%)	SEM	MD	CC(r^2^); p* value
S-WBV 30Hz/2mm		band-stop filter						
MNF(Hz)	1	86.81 (±23.05)	0.99	0.98–1	3.38	2.73	7.54	0.99; 0.000001
	2	84.86 (±19.97)						
MNF(Hz)	3	89.38 (±26.24)	0.99	0.98–1	3.15	3.01	8.31	0.99; 0.00001
	4	89.3 (±29.85)						
MDF(Hz)	1	69.66 (±18.39)	0.99	0.97–1	3.99	2.72	7.52	0.97; 0.000007
	2	68.21 (±15.82)						
MDF(Hz)	3	72.08 (±24.45)	0.98	0.97–1	5.22	4.02	11.12	0.99; 0.000001
	4	72.37 (±29.69)						
EMG RMS (%MVC)	1	20.32 (±8.15)	0.89	0.74–1	15.62	3.24	8.96	0.69; 0.01
	2	19.21 (±6.34)						
EMG RMS (%MVC)	3	20.13 (±8.15)	0.98	0.95–1	11.39	2.02	5.57	0.96; 0.00001
	4	22.28 (±10.35)						
**S-WBV 30Hz/2mm**		**high-pass filter**						
MNF(Hz)	1	189.78 (±15.18)	0.94	0.85–1	2.52	5.07	14.01	0.78; 0.003
	2	189.03 (±14.36)						
MNF(Hz)	3	189.87 (±14.16)	0.97	0.94–1	2.12	3.58	9.90	0.98; 0.00001
	4	193.13 (±18.75)						
MDF(Hz)	1	164.29 (±14.65)	0.98	0.95–1	1.70	2.74	7.57	0.93; 0.0001
	2	162.75 (±13.58)						
MDF(Hz)	3	165.46 (±13.25)	0.96	0.92–1	2.63	4.04	11.17	0.96; 0.00001
	4	168.62 (±18.15)						
EMG RMS (%MVC)	1	10.23 (±4.29)	0.92	0.81–1	15.93	1.45	4.01	0.80; 0.002
	2	9.18 (±3.11)						
EMG RMS (%MVC)	3	10.14 (±3.59)	0.95	0.88–1	12.23	1.21	3.35	0.83; 0.001
	4	11.02 (±4.16)						

Note: p*CC with significance

Abbreviations: ICC, interclass correlation coefficient model 3,3 (for three repetitions of sEMG recording); CI, confidence interval; CV, coefficient of variation; SEM, standard error of measurement (units as for measurement); MD, minimal differences (units as for measurement); CC, coefficient of correlations with significance (p value); EMG, surface electromyography; MNF, mean frequency; MDF, median frequency; EMG RMS %MVC, the mean of root mean square of surface electromyography normalized to MVC.

When band-stop filtered, the mean normalized sEMG_RMS_ amplitude, mean and median frequencies during vibration intensity of 40 Hz/4mm showed substantial intraday reliability. The inter-measurement variability was between 2.81% and 11.75%. The ICC for high-pass filtered mean and median frequencies during the 40Hz/4mm vibration was indicative of substantial reproducibility. The inter-measurement variability ranged between 1.90% and 22.89% (Table 3).

**Table 3:** Band-stop filtered and high-pass filtered intraday reliability of 40Hz/4mm—whole body vibration sEMG signals from the pelvic floor muscle.

Parameter (unit)	session	mean (±SD)	ICC	95% CI	CV (%)	SEM	MD	CC (r^2^); p* value
S-WBV 40Hz/4mm		band-stop filter						
MNF(Hz)	1	92.43 (±22.79)	0.99	0.98–1	4.19	3.25	8.97	0.97; 0.00007
	2	89.20 (±25.06)						
MNF(Hz)	3	91.76 (±21.94)	0.99	0.98–1	2.81	2.73	7.55	0.99; 0.00002
	4	91.62 (±25.47)						
MDF(Hz)	1	74.66 (±19.70)	0.97	0.94–1	6.42	4.70	12.99	0.91; 0.0002
	2	72.25 (±21.64)						
MDF(Hz)	3	76.04 (±21.06)	0.99	0.98–1	3.32	2.54	7.03	0.98;0.0001
	4	75.04 (±23.93)						
EMG RMS (%MVC)	1	33.87 (±14.09)	0.95	0.89–1	11.75	3.66	10.11	0.88; 0.005
	2	31.42 (±11.29)						
EMG RMS (%MVC)	3	29,89 (±10.69)	0.97	0.93–1	8.97	2.74	7.57	0.89; 0.0004
	4	31.28 (±11.75)						
**S-WBV 40Hz/4mm**		**high-pass filter**						
MNF(Hz)	1	186.19 (±15.96)	0.97	0.94–1	1.90	3.78	10.46	0.91; 0.0002
	2	186.26 (±17.90)						
MNF(Hz)	3	187.56 (±15.13)	0.92	0.83–1	4.11	7.51	20.74	0.92; 0.0001
	4	192.36 (±24.22						
MDF(Hz)	1	161.97 (±15.58)	0.98	0.96–1	1.94	3.27	9.03	0.95; 0.00004
	2	163.12 (±18.11)						
MDF(Hz)	3	164.37 (±13.89)	0.88	0.74–1	5.52	9.15	25.27	0.90; 0.0003
	4	169.21 (±25.36)						
EMG RMS (%MVC)	1	18.20 (±9.71)	0.92	0.82–1	22.89	3.20	8.84	0.82; 0.002
	2	14.92 (±6.86)						
EMG RMS (%MVC)	3	14.25 (±4.93)	0.97	0.94–1	8.42	1.22	3.38	0.92; 0.0002
	4	14.88 (±5.71)						

Note: p*CC with significance

Abbreviations: ICC, interclass correlation coefficient model 3,3 (for three repetitions of sEMG recording); CI, confidence interval; CV, coefficient of variation; SEM, standard error of measurement (units as for measurement); MD, minimal differences (units as for measurement); CC, coefficient of correlations with significance (p value); EMG, surface electromyography; MNF, mean frequency; MDF, median frequency; EMG RMS %MVC, the mean of root mean square of surface electromyography normalized to MVC.

### Interday reliability

The ICCs for band-stop filtered mean and median frequency of the 30Hz/2mm and 40Hz/4mm S-WBV sessions were indicative of fair reliability. The variability between the measurements showed values between 17.10% and 22.28% for measured variables. The interday reliability (session 1 vs. session 3) of the mean normalized sEMG_RMS_ amplitude for band-stop filtered means of 40 Hz/4 mm and 30Hz/2mm vibration recordings was substantial. The correlation coefficient was only significant for the mean normalized amplitude (Table 4).

**Table 4:** Band-stop filtered and high-pass filtered interday reliability of 30Hz/2mm and 40Hz/4mm—whole body vibration sEMG signals from the pelvic floor muscle.

Parameter (unit)/ intensity of vibration	session	mean (±SD)	ICC	95% CI	CV (%)	SEM	MD	CC (r^2^); p value
		band-stop filter						
MNF(Hz)/ 30Hz/2mm	1	86.81 (±23.5)	0.59	0.14–1	20.10	18.83	52.03	0.18; 0.2
	3	89.38 (±26.24)						
MNF(Hz)/ 40Hz/4mm	1	92.43 (±22.79)	0.53	0.06–1	19.14	18.91	52.26	0.13; 0.4
	3	91.76 (±21.94						
MDF(Hz)/ 30Hz/2mm	1	69.67 (±18.39)	0.55	0.06–1	17.10	16.7	46.12	0.15; 0.3
	3	72.08 (±24.45)						
MDF(Hz)/ 40Hz/4mm	1	74.67 (±19.70)	0.43	0.02–0.99	22.28	18.00	49.76	0.11; 0.4
	3	76.04 (±21.06)						
EMG RMS %MVC/ 30Hz/2mm	1	20.26 (±8.22)	0.93	0.84–1	13.83	2.98	8.23	0.75; 0.005*
	3	20.06 (±8.23)						
EMG RMS %MVC/ 40Hz/4mm	1	33.87 (±14.09)	0.82	0.59–1	22.27	6.96	19.23	0.55; 0.03*
	3	29.89 (±10.69)						
		**high-pass filter**						
MNF(Hz)/ 30Hz/2mm	1	189.78 (±15.18)	0.56	0.08–1	5.66	11.49	31.75	0.15; 0.3
	3	189.87 (±14.16)						
MNF(Hz)/ 40Hz/4mm	1	186.19 (±15.96)	0.35	0–0.96	6.91	13.78	38.07	0.04; 0.6
	3	187.56 (±15.13)						
MDF(Hz)/ 30Hz/2mm	1	164.29 (±14.65)	0.24	0–0.89	7.37	12.96	35.82	0.02; 0.7
	3	165.46 (±13.25						
MDF(Hz)/ 40Hz/4mm	1	161.97 (±15.58)	0.16	0–0.83	8.14	14.09	38.93	0.01; 0.8
	3	164.37 (±13.89)						
EMG RMS %MVC/ 30Hz/2mm	1	10.24 (±4.29)	0.90	0.77–1	15.56	1.69	4.68	0.65; 0.01*
	3	10.14 (±3.59)						
EMG RMS %MVC/ 40Hz/4mm	1	18.19 (±9.71)	0.73	0.41–1	33.79	5.04	13.94	0.50; 0.04*
	3	14.25 (±4.93)						

Note: p*CC with significance

Abbreviations: ICC, interclass correlation coefficient model 3,3 (for three repetitions of sEMG recording); CI, confidence interval; CV, coefficient of variation; SEM, standard error of measurement (units as for measurement); MD, minimal differences (units as for measurement); CC, coefficient of correlations with significance (p value); EMG, surface electromyography; MNF, mean frequency; MDF, median frequency; EMG RMS %MVC, the mean of root mean square of surface electromyography normalized to MVC.

The ICCs for high-pass filtered mean and median frequencies of the 30Hz/2mm and 40/4mm S-WBV sessions indicated slight to fair reproducibility. The variability between the measurements showed values between 5.66% and 8.14% for measured variables. The interday reliability of the mean normalized amplitude for high-pass filter at 100-450Hz was substantial for the 30Hz/2mm S-WBV and moderate for the 40/4mm S-WBV. The respective correlation coefficients were significant (Table 4).

### The intra- and interday reliability of MVC

The intra- and interday reliability of the greatest value and average MVC amplitude exhibited substantial reproducibility (Table 5).

**Table 5:** Intraday and interday reliability of pelvic floor muscle MVC.

Parameter (unit)	session	mean (±SD)	ICC	95% CI	CV (%)	SEM	MD	CC (r^2^); p value
MVC^B^ (μV)	1	49.98 (±15.41)	0.83	0.62–1	11	5.71	15.78	0.73; 0.007
	2	52.48 (±12.69)						
MVC^B^ (μV)	3	55.71 (±19.34)	0.98	0.93–1	9.94	3.32	9.17	0.94; 0.0006
	4	49.74 (±18.68)						
MVC^B^ (μV)	1	49.98 (±15.41	0.84	0.63–1	14.52	6.97	19.26	0.74; 0.005
	3	55.71 (±19.34)						
MVC^A^ (μV)	1	45.71 (14.17)	*0.95	0.88–1	8.53	4.15	11.46	0.83; 0.001
	2	46.65 (13.14)						
MVC^A^ (μV)	3	53.40 (19.85)	*0.94	0.88–1	0.14	5.73	15.83	0.84; 0.001
	4	46.21 (16.91)						
MVC^A^ (μV)	1	45.71 (14.17)	*0.82	0.58–1	21.21	9.61	26.55	0.53; 0.04
	3	53.40 (19.85)						

Note: p*CC with significance

Abbreviations: ICC, interclass correlation coefficient model 3,1 (for the greatest value of three MVC trials)

*ICC, interclass correlation coefficient model 3,3 (for three trials of MVC); CI, confidence interval; CV, coefficient of variation; SEM, standard error of measurement (units as for measurement); MD, minimal differences (units as for measurement); CC, coefficient of correlations with significance (p value); MVC^B^ the greatest value of the three trials of maximal voluntary contraction, MVC^A^ average maximal voluntary contraction (mean amplitude of three MVC trials).

## Discussion

Surface electromyography is commonly used in vibration studies. Since vibrations inevitably contaminate the surface EMG signal with motion artifacts, a number of filtering regimens are recommended to remove the vibration frequency [17–19]. However, it should also be considered that filtering partly modifies sEMG signals and may therefore affect result interpretation. Deleting the spikes in the sEMG spectrum might not only eliminate the artifacts, but also remove parts of reflex activity evoked by vibration [16]. When high-pass filter is used, a loss in sEMG activity is observed as the entire frequency spectrum below 100Hz becomes attenuated [18]. In turn, it has been pointed out that the band-stop filter progressively underestimates the sEMG_RMS_ during WBV [20].

The authors of the few studies exploring the effects of whole body vibration on PFM activity in healthy individuals and/or those with PFM dysfunction used sEMG signal filtering to remove artifacts. In the study of Luginbuehl et al. [28], the fundamental frequency and harmonic content of the stochastic resonance WBV EMG’s raw signal parts were spectrum analyzed by Fast Fourier Transformation (FFT) and removed by notch filtering. For EMG signals, Lee et al. [12] used 80~250 Hz of high-pass filter to remove noises, and subsequently calculated the root mean square value.

It is still undetermined whether PFM sEMG obtained using sEMG signal filtering methods have satisfactory reliability. To our knowledge, this is the first report to evaluate reliability of PFM sEMG during vibration after signal processing with band-stop and high-pass at 100-450Hz filters. Hence, no comparisons can be made regarding the levels of reliability, CV%, SEM and MD values. We evaluated sEMG variables (the mean normalized amplitude, mean frequency and median values) during whole body vibration of two intensities. The intraday ICC for band-stop and high-pass filtered mean and median frequencies and the mean normalized sEMG_RMS_ amplitude of 30Hz/2mm vibration indicated substantial reliability. The intraday reliability of band-stop filtered and high-pass filtered PFM sEMG variables of 40Hz/4mm intensity vibration was also substantial.

High-pass filtered interday sEMG recordings of the 40Hz/4mm measurement were slightly reproducible for MNF and MDF (ICCs of 0.35 and 0.16, respectively), and moderately reproducible with respect to the mean sEMG_RMS_ %MVC amplitude (ICC = 0.73). The interday reliability of the high-pass filtered 30Hz/2mm vibration recordings was somewhat higher for MNF and MDF (ICC of 0.56 and 0.24, respectively) and substantial for the mean sEMG_RMS_ %MVC amplitude (ICC = 0.90). The ICCs for band-stop filtered MNF and MDF of the 30Hz/2mm and 40Hz/4mm S-WBV sessions were indicative of fair reliability (ICC of 0.43 to 0.59). The interday reliability of the mean sEMG_RMS_ %MVC amplitude for band-stop filtered 40 Hz/4mm and 30Hz/2mm vibration recordings was substantial (ICC of 0.82 and 0.93 respectively).

The intraday reliability of the mean normalized amplitude EMG_RMS_ %MVC of the 30Hz/2mm and 40Hz/4mm S-WBV sessions was substantial while the interday (test-retest) reliability was moderate to substantial for both signal filtering methods. Our study demonstrated that the intraday sEMG reliability of the MNF and MDF ranged from moderate to substantial while the interday reliability was poor.

An analysis of PFM MVC reliability revealed some similarities between our and other authors’ findings. Our study showed substantial intraday and interday reliability of the MVC. Grape et al. [14] obtained good to high reliability and demonstrated that choosing the highest contraction resulted in slightly higher ICCs compared to the mean of three contractions. There are some differences between these two studies though, i.e., the MVC times were 10 and 5 seconds in the study of Grape et al. and ours, respectively. Also, vaginal probes were different (pear-shaped vs. longitudinal). Scharschmidt et al. [29] used sEMG probes with a circumferential electrode position; the intraday and interday reliability (reproducibility) of PFM MVC was moderate. A pear-shaped probe was used by Koenig et al. [13]; the inter- and intraday PFM sEMG reliability was moderate and relatively high, respectively. Auchincloss and McLean [30] demonstrated that although the between-trial sEMG reliability was fair to high, the interday reliability was poor. Lower interday compared to intraday ICCs of sEMG recordings from the PFM might be associated with the fact that vaginal surface electrodes cannot be fixed directly onto the pelvic muscle; minimal displacement during vibration is therefore possible. We are aware that comparisons of PFM sEMG recordings pose problems related to tissue hydration and temperature inside the vagina, menstruation cycle and potential crosstalk from other muscles. In order to monitor abdominal muscle coactivation, sEMG feedback was used during all trials.

The parameters of synchronous vibration applied in our study had been determined in a pilot experiment. Stania et. al. [11] revealed that high-intensity whole body vibrations (40Hz/4mm) of long duration (60s, 90s) increased the mean amplitude of sEMG signal from the PFM in young continent women, and did not cause pelvic floor muscle fatigue. We therefore used 60-second synchronous whole body vibration (Fitvible 600) of two intensities: frequency/ amplitude: 30 Hz/2mm and 40 Hz/4 mm.

The activity of PFM depends on body positions; the level of background muscle activity increases in standing [31, 32]. In the present study, the MVC and non- vibration and vibration exposures were examined in the standing position with the knee and hip joints bent at 35° [11] and arms hanging loosely. Lauper et al.’s [10] participants stood on the vibration platform with slightly bent knees and neutral hip position during vibration (stochastic resonance WBV and sinusoidal vibrations). Other researchers found that a 40° knee flexion caused an increase in PFM activation during side-alternating vibration (Galileo) [12]. Still, others pointed out that a knee flexion angle of 26–30° significantly reduced the adverse effects through a decrease in vibration transmissibility to the head and the upper body; they also suggested that the use of small knee flexion angles (10–15°) during WBV increased the likelihood of negative side effects and should be avoided [33]. Another issue to consider is enhancement of reflex muscle responses to vibration by muscular contractions [6]. We could not be sure whether the pelvic floor muscles would contract with the same intensity during all vibration sessions. Therefore, similar to Luginbuehl et al. [28], we decided not to ask the participants to contract PFM during vibration. WBV has been recognized as beneficial in the management of PFM dysfunction [34]; hence a need for further studies on changes in pelvic floor muscle sEMG during whole body vibration both in healthy women and those with PFM dysfunction.

### Limitations

Some limitations of the study should be noted. sEMG recordings were performed in healthy women so the reliability data cannot apply to individuals with pelvic floor muscle dysfunction. Also, three women suffered acute effects of high intensity vibration including erythema and itching; the experienced discomfort might have affected sEMG results. No studies could be found to confirm reliability of results obtained with a pear-shaped electrode which we used; it should be noted though that Scharschmidt et al. [29] concluded that electrode arrangement (longitudinal vs circumferential) had no effect on the reliability of sEMG data. Finally, the knee angle was only controlled with a goniometer.

## Conclusions

Our study was focused on the assessment of intraday and interday reliability of reflex PFM sEMG activity during 60 seconds of synchronous whole-body vibration of two intensities (30Hz/2mm; 40Hz/4mm) using signal processing methods described in literature (band-stop filter, high-pass filter).

Band-stop filtering and high-pass filtering of the mean normalized amplitude obtained from three PFM sEMG recordings made during S-WBV of two intensities yielded intraclass correlation coefficients indicating substantial intraday reliability while the interday reliability was moderate to substantial. Despite slight differences, SEM, CV% and MD of sEMG_RMS_ %MVC were not high for both filtering methods. The intraday reliability of MNF and MDF reached substantial reproducibility for band-stop and high-pass filtering. However, the ICCs for MNF and MDF cover a broad range of interday reliability (from slight to fair) while SEM and MD are high. The intraday reliability proved satisfactory for all variables; however, the interday comparison showed moderate to substantial ICC only for the mean sEMG_RMS_ amplitude. We therefore recommend this parameter should be used when analyzing PFM sEMG recorded during vibration. The intra- and interday reliability of the greatest value and average MVC amplitude exhibited substantial reproducibility.

Our study showed similar reliability of PFM sEMG during S-WBV in case of the two filtering methods used. Therefore, the question arises as to whether it is possible to compare results from different research centers which use different filtering methods of sEMG recorded during WBV. Our results indicate a need for further interpretations of PFM sEMG recordings obtained during S-WBV for the needs of clinical studies in patients with PFM dysfunctions.

## Supporting information

S1 Dataset Study dataset. (XLSX)

## References

[1] Centner C, Ritzmann R, Gollhofer A, König D. Effects of Whole-Body Vibration Training and Blood Flow Restriction on Muscle Adaptations in Women: A Randomized Controlled Trial. J Strength Cond Res. 2020;34(3):603–8. 10.1519/JSC.0000000000003401 31842133

[2] Alam MM, Khan AA, Farooq M. Effect of whole-body vibration on neuromuscular performance: A literature review. Work 2018;59(4):571–83. 10.3233/WOR-182699 29733043

[3] Stania M, Juras G, Słomka K, Chmielewska D, Król P. The Application of Whole-Body Vibration in Physiotherapy—A Narrative Review Physiol Int. 2016; 1;103(2):133–145. 10.1556/036.103.2016.2.1 28639859

[4] Dionello CF, de Souza PL, Sá-Caputo D, Morel DS, Moreira-Marconi E, Paineiras-Domingos LL, et al. Do whole body vibration exercises affect lower limbs neuromuscular activity in populations with a medical condition? A systematic review. Restor Neurol Neurosci. 2017; 35(6):667–81. 10.3233/RNN-170765 29172012

[5] Rauch F, Sievanen H, Boonen S, Cardinale M, Degens H, Felsenberg D, et al. International Society of Musculoskeletal and Neuronal Interaction. Reporting whole-body vibration intervention studies: recommendations of the International Society of Musculoskeletal and Neuronal Interactions. J Musculoskelet Neuronal Interact. 2010;10(3):193–8. 20811143

[6] Eklund G, Hagbarth K. Normal variability of tonic vibration reflex in man. Exp Neurol. 1966;16:80–92. 10.1016/0014-4886(66)90088-4 5923486

[7] De Gail P, Lance JW, Neilson PD. Differential effects on tonic and phasic reflex mechanisms produced by vibration of muscles in man. J Neurol Neurosurg Psychiat. 1966; 29(1):1–11. 10.1136/jnnp.29.1.1 5910574 PMC495977

[8] Cochrane DJ. Vibration Exercise: The Potential Benefits. Int J Sports Med. 2011;32(2):75–99. 10.1055/s-0030-1268010 21165804

[9] Vodusek D.B. Anatomy and neurocontrol of the pelvic floor. Digestion 2004;69(2):87–92. 10.1159/000077874 15087575

[10] Lauper M, Kuhn A, Gerber R, Luginbühl H, Radlinger L. Pelvic floor stimulation: what are the good vibrations? Neurourol Urodyn. 2009;5:405–10. 10.1002/nau.20669 19283866

[11] Stania M, Chmielewska D, Kwaśna K, Smykla A, Taradaj J, Juras G.Bioelectrical Activity of the Pelvic Floor Muscles During Synchronous Whole-Body Vibration—A Randomized Controlled Study. BMC Urol 2015 10 24;15:107. 10.1186/s12894-015-0103-9 26498430 PMC4619551

[12] Lee J, Lee K, Song C. Determining the Posture and Vibration Frequency that Maximize Pelvic Floor Muscle Activity During Whole-Body Vibration. Med Sci Monit. 2016;22:4030–6. 10.12659/msm.898011 27787476 PMC5087668

[13] Koenig I, Luginbuehl H, Radlinger L. Reliability of pelvic floor muscle electromyography tested on healthy women and women with pelvic floor muscle dysfunction. Ann Phys Rehabil Med. 2017;60(6):382–86. 10.1016/j.rehab.2017.04.002 28716538

[14] Grape HH, Dedering A, Jonasson AF. Retest reliability of surface electromyography on the pelvic floor muscles. Neurourol Urodyn. 2009;28(5):395–9. 10.1002/nau.20648 19214991

[15] Fratini A, Cesarelli M, Bifulco P, Romano M. Relevance of motion artifact in electromyography recordings during vibration treatment. J Electromyogr Kinesiol. 2009;19(4):710–8. 10.1016/j.jelekin.2008.04.005 18495492

[16] Lienhard K, Cabasson A, Meste O, Colson SS. sEMG during Whole-Body Vibration Contains Motion Artifacts and Reflex Activity. J Sports Sci Med. 2015a;14(1):54–61. eCollection 2015 Mar. 25729290 PMC4306783

[17] Sebik O, Karacan I, Cidem M, Türker KS. Rectification of SEMG as a tool to demonstrate synchronous motor unit activity during vibration. J Electromyogr Kinesiol. 2013;23(2):275–84. 10.1016/j.jelekin.2012.09.009 23098913

[18] Hazell Tom J, Kenno Kenji A, Jakobi Jennifer M. Evaluation of Muscle Activity for Loaded and Unloaded Dynamic Squats during Vertical Whole-Body Vibration. J Strength Cond Res. 2010; 24(7):1860–1865. 10.1519/JSC.0b013e3181ddf6c8 20543737

[19] Abercromby AF, Amonette WE, Layne CS, McFarlin BK, Hinman MR, Paloski WH. Variation in neuromuscular responses during acute whole-body vibration exercise. Med Sci Sports Exerc. 2007a; 39(9): 1642–50. 10.1249/mss.0b013e318093f551 17805098

[20] Lienhard K, Cabasson A, Meste O, Colson SS. Comparison of sEMG processing methods during whole-body vibration exercise. J Electromyogr Kinesiol. 2015b;25(6):833–40. 10.1016/j.jelekin.2015.10.005 26565598

[21] Ritzmann R, Kramer A, Gruber M, Gollhofer A, Taube W. EMG activity during whole body vibration: motion artifacts or stretch reflexes? Eur J Appl Physiol. 2010;110(1):143–51. 10.1007/s00421-010-1483-x 20419311

[22] Walter SD, Eliasziw M, Donner A. Sample size and optimal designs for reliability studies. Statistics in Medicine. 1998. 17: 101–110. 10.1002/(sici)1097-0258(19980115)17:1&lt;101::aid-sim727&gt;3.0.co;2-e 9463853

[23] Micussi MT, Freitas RP, Angelo PH, Soares EM, Lemos TM, Maranhão TM, Is there a difference in the electromyographic activity of the pelvic floor muscles across the phases of the menstrual cycle? J Phys Ther Sci. 2015;27(7):2233–2237. 10.1589/jpts.27.2233 26311960 PMC4540855

[24] Keshwani N, McLean L. State of the art review: Intravaginal probes for recording electromyography from the pelvic floor muscles. Neurourol Urodyn. 2015 2;34(2):104–12. 10.1002/nau.22529 24264797

[25] Koo TK, Li MY. A Guideline of Selecting and Reporting Intraclass Correlation Coefficients for Reliability Research. Chiropr Med. 2016;15(2):155–163. 10.1016/j.jcm.2016.02.012 27330520 PMC4913118

[26] Shrout PE. Measurement reliability and agreement in psychiatry. Stat Methods Med Res. 1998 9;7(3):301–17. 10.1177/096228029800700306 9803527

[27] Weir J.P. Quantifying test-retest reliability using the intraclass correlation coefficient and the SEM. J Strength Cond Res. 2005;19(1):231–40. 10.1519/15184.1 15705040

[28] Luginbuehl H, Lehmann C, Gerber R, Kuhn A, Hilfiker R, Baeyens J.P., et al. Continuous versus intermittent stochastic resonance whole body vibration and its effect on pelvic floor muscle activity. Neurourol Urodyn 2012; 31(5):683–7, 10.1002/nau.21251 22395850

[29] Scharschmidt R, Derlien S, Siebert T, Herbsleb M, Stutzig N. Intraday and interday reliability of pelvic floor muscles electromyography in continent woman. Neurourol Urodyn. 2020;39(1):271–278. 10.1002/nau.24187 31642114

[30] Auchincloss CC, McLean L. The reliability of surface EMG recorded from the pelvic floor muscles. J Neurosci Methods. 2009;182(1):85–96. 10.1016/j.jneumeth.2009.05.027 19539646

[31] Chmielewska D, Stania M, Sobota G, Kwaśna K, Błaszczak E, Taradaj J, et al. Impact of different body positions on bioelectrical activity of the pelvic floor muscles in nulliparous continent women. Biomed Res Int. 2015;2015:905897. 10.1155/2015/905897 25793212 PMC4352464

[32] Lee K. Investigation of Electromyographic Activity of Pelvic Floor Muscles in Different Body Positions to Prevent Urinary Incontinence. Med Sci Monit. 2019; 25: 9357–63. 10.12659/MSM.920819 31813929 PMC6918805

[33] Abercromby AFJ, Amonette WE, Layne CS, McFarlin BK, Hinman MR, Paloski WH. Vibration exposure and biodynamic responses during whole-body vibration training. Med Sci Sports Exerc. 2007b;39(10):1794–800. 10.1249/mss.0b013e3181238a0f 17909407

[34] Guedes-Aguiar EO, de Sá-Caputo DDC, Moreira-Marconi E, de Macêdo Uchôa SM, de Barros PZ, Valentin EK, et al. Effect of whole-body vibration exercise in the pelvic floor muscles of healthy and unhealthy individuals: a narrative review. Transl Androl Urol. 2019;8(4):395–404. 10.21037/tau.2019.06.14 31555564 PMC6732087

